# Development and Application of an In Vitro Drug Screening Assay for *Schistosoma mansoni* Schistosomula Using YOLOv5

**DOI:** 10.3390/biomedicines12122894

**Published:** 2024-12-19

**Authors:** María Alejandra Villamizar-Monsalve, Javier Sánchez-Montejo, Julio López-Abán, Belén Vicente, Miguel Marín, Noelia Fernández-Ceballos, Rafael Peláez, Antonio Muro

**Affiliations:** 1 Infectious and Tropical Diseases Research Group (e-INTRO), Biomedical Research Institute of Salamanca, Research Centre for Tropical Diseases at the University of Salamanca (IBSAL-CIETUS), Faculty of Pharmacy, University of Salamanca, 37007 Salamanca, Spain; mavillamizar@usal.es (M.A.V.-M.); s.montejo@usal.es (J.S.-M.); belvi25@usal.es (B.V.); ama@usal.es (A.M.); 2 Organic and Pharmaceutical Chemistry Department, Biomedical Research Institute of Salamanca Research (IBSAL), Faculty of Pharmacy, University of Salamanca, 37007 Salamanca, Spain; mmarin@usal.es (M.M.); nferceb@usal.es (N.F.-C.); pelaez@usal.es (R.P.)

**Keywords:** schistosomiasis, *Schistosoma mansoni*, schistosomula, drug screening, artificial intelligence

## Abstract

Background: Schistosomiasis impacts over 230 million people globally, with 251.4 million needing treatment. The disease causes intestinal and urinary symptoms, such as hepatic fibrosis, hepatomegaly, splenomegaly, and bladder calcifications. While praziquantel (PZQ) is the primary treatment, its effectiveness against juvenile stages (schistosomula) is limited, highlighting the need for new therapeutic agents, repurposed drugs, or reformulated compounds. Existing microscopy methods for assessing schistosomula viability are labor-intensive, subjective, and time-consuming. Methods: An artificial intelligence (AI)-assisted culture system using YOLOv5 was developed to evaluate compounds against *Schistosoma mansoni* schistosomula. The AI model, based on object detection, was trained on 4390 images distinguishing between healthy and damaged schistosomula. The system was externally validated against human counters, and a small-scale assay was performed to demonstrate its potential for larger-scale assays in the future. Results: The AI model exhibited high accuracy, achieving a mean average precision (mAP) of 0.966 (96.6%) and effectively differentiating between healthy and damaged schistosomula. External validation demonstrated significantly improved accuracy and counting time compared to human counters. A small-scale assay was conducted to validate the system, identifying 28 potential compounds with schistosomicidal activity against schistosomula in vitro and providing their preliminary LC_50_ values. Conclusions: This AI-powered method significantly improves accuracy and time efficiency compared to traditional microscopy. It enables the evaluation of compounds for potential schistosomiasis drugs without the need for dyes or specialized equipment, facilitating more efficient drug assessment.

## 1. Introduction

Schistosomiasis is considered one of the 20 neglected tropical diseases (NTDs), affecting more than 230 million people worldwide and accounting for 1.9 million disability-adjusted life years (DALYs) in more than 78 countries at risk of infection. In 2021, the WHO estimated that at least 251.4 million people, mainly in Sub-Saharan Africa, needed treatment for schistosomiasis [1]. It is caused by digenetic trematodes of the genus *Schistosoma* spp., with *S. mansoni*, *S. haematobium*, *S. japonicum*, *S. mekongi*, and *S. intercalatum* standing out as the species with the most significant implications in humans. Each species’ distribution depends on the habitat of the intermediate host snails [2,3]. *S. japonicum* infections may induce Katayama fever, with patent infections exhibiting intestinal symptoms (in *S. mansoni* and *S. japonicum* infections) along with urinary symptoms. Chronic infection can result in periportal liver fibrosis and hepatosplenomegaly, while *S. haematobium* infections are linked to bladder calcification and genitourinary complications [4]. Their current treatment of choice is praziquantel (PZQ), a pyrazinoisoquinoline introduced in the 1980s. However, PZQ only kills adult worms and poorly affects juvenile stages, known as schistosomula, which are the larval form that develops after cercariae penetrate the host’s skin and lose their tails [5]. Since control programs rely solely on the mass administration of PZQ, they often fail not only due to its limited ability to interrupt the disease but also because global coverage remains low, reaching only 31.9% of the population in need of treatment [6]. Furthermore, the repeated use of this drug in mass chemotherapy and treatment campaigns raises concerns about the development of drug resistance [7,8]. Additionally, addressing schistosomiasis presents a significant challenge due to the long-term persistence of active infections and the high frequency of reinfections. The overall mean reinfection rate for schistosomiasis is notably high, at 36.1% [9]. Given these concerns, researchers are designing new targets and exploring molecules licensed for other uses that could target both adult and schistosomula stages of *Schistosoma* spp. [10,11]. Various techniques are employed to assess the viability of schistosomula. Staining methods like methylene blue and toluidine blue differentiate between viable and nonviable phenotypes [12]. Fluorescence-based assays use compounds such as fluorescein diacetate (FDA) and propidium iodide (PI). Additionally, viability is assessed through techniques like ATP quantification, using dyes such as resazurin [13,14], and colorimetric MTT quantitative assays [15]. Qualitative methods evaluate changes in motility, categorizing phenotypic characteristics through microscopic observation to distinguish between healthy, damaged, or death stages [16]. Confocal laser scanning microscopy is also employed for detailed examination [17]. Artificial intelligence (AI) has recently been applied to detect and screen various parasites, including helminths, mainly through object detection techniques. For instance, it has been utilized for automated scanning and detection of helminth eggs from soil-transmitted helminths such as *Ascaris lumbricoides*, *Trichuris trichiura*, hookworms, and *S. mansoni* [18,19]. Additionally, AI has been used to assess viability from morphological and motility perspectives in various helminths, including nematodes like *Brugia malayi* and *Dirofilaria immitis* [20]. AI has also been employed to identify *Schistosoma* spp. intermediate hosts and the parasite itself [21]. These methods have also evaluated *Schistosoma* spp. adults and schistosomula. An in vitro experimental assessment was conducted using automated imaging of both stages of *S. mansoni*, although these are not publicly accessible [22,23]. Various artificial intelligence programs, such as Roboflow (https://roboflow.com), are available for free use, which aims to accelerate and simplify the process of labeling images and training different models [24]. YOLOv5 (You Only Look Once) is another software using an object detection algorithm that divides images into a grid system, where each grid cell detects objects independently. YOLOv5 supports model training and retraining through detection and labeling verification, and it is compatible with platforms like Google Colab and Kaggle, which provide CUDA/CUDNN, Python, and PyTorch environments [25]. In parasitology, YOLOv5 has been employed to detect parasitic eggs in human feces [26] and classify species of *Plasmodium falciparum*, often integrated with other algorithms like DarkNet-53 [27].

This study aimed to establish and refine a semi-automated method for assessing the efficacy of antiparasitic compounds against *S. mansoni* schistosomula using YOLOv5. This method seeks to facilitate the screening of compounds and could be adapted for larger-scale assays in the future, integrating microscopy and AI to improve drug assessment for schistosomiasis.

## 2. Materials and Methods

### 2.1. Maintenance of S. mansoni Life Cycle and Experimental Animals

*S. mansoni* life cycle was maintained in the laboratory following the protocol by Vicente et al. [28]. The procedure involved handling mice under Spanish regulations (RD53/2013) and European Union guidelines (Di 2012/63/EU). The Research Ethics Committee of the University of Salamanca (Spain) approved all procedures for this study (Protocols: CEI 1062 and CEI 1080). CD1 mice aged 6–8 weeks, sourced from Charles Rivers Laboratories (Lyon, France), were housed in polycarbonate and wire cages with ad libitum access to food and water under a 12 h light–dark cycle at a temperature of 22–25 °C. Cercariae were obtained from *Biomphalaria glabrata* snails previously infected with 7–10 miracidia. After 28 days, the snails were exposed to light at a temperature of 26 °C for 2.5 to 3 h to induce cercarial emission, which were then collected and counted.

### 2.2. Cercaria Transformation, Schistosomula In Vitro Culture, and Compound Screening

Cercaria transformation, schistosomula in vitro culture, and compound screening were adapted from protocols by Tucker et al. [29] and Lombardo et al. [30]. After obtaining the cercariae suspension, they were immobilized on ice for 30 min. Coarse residues were then removed using a pipette. The suspension was centrifuged at 200× *g* for 3 min at room temperature in a 50 mL tube, and the supernatant was carefully removed. Hanks’ Balanced Salt Solution (HBSS) was added, supplemented with penicillin/streptomycin (100 units/100 μg/mL at 4 °C) if necessary. This suspension was transferred to a 10 mL Luer-lock syringe connected via a three-way Luer-lock stopcock to another syringe of the same volume. The suspension was then alternated between the syringes 5 to 10 times. Then, 50 μL of suspension containing 30 to 40 schistosomula was placed in a 96-well plate with a curved bottom. Afterward, 175 μL of phenol red-free Dulbecco’s Modified Eagle Medium (DMEM) supplemented with penicillin and streptomycin (100 units/100 μg/mL at 37 °C) was added to each well. The outer wells of the 96-well plate were excluded to prevent edge effects and evaporation. The plate was then incubated at 37 °C in a 5% CO_2_ environment overnight to ensure complete transformation of the schistosomula. Since PZQ activity has been reported to be less effective against schistosomula, other antiparasitic commercial drugs were screened as controls (ivermectin, albendazole, mebendazole, auranofin, and triclabendazole sulfoxide). Also, DMEM with 1% dimethyl sulfoxide (DMSO) was used as a basal control. Screening procedures were carried out at a final drug concentration of 10 µM. After adding compounds, plates were centrifuged at 200× *g* for 2 min to ensure all schistosomula settled at the well bottom; then, plates were incubated at 37 °C in a 5% CO_2_ environment. Bottom well images at 20× magnification were captured at 0 h, 24 h, 48 h, and 72 h after treatment. All treatment conditions were conducted in triplicate and repeated at least three times. The viability percentage was calculated using the formula: Viability Percentage = (number of healthy schistosomula at 72 h/number of healthy schistosomula at hour 0) × 100. The inhibition percentage was calculated using the formula: Inhibition Percentage = (1 − (viability percentage of sample/viability percentage of control)) × 100. The concentration at which 50% of schistosomula are classified as “damaged” (characterized by an irregular shape, granularity, and dark coloration indicative of tegument and internal damage) was defined as the lethal concentration of 50% (LC_50_). This value was calculated using a three-parameter logistic model (LL.3) within a dose–response model. Active molecules were tested at concentrations of 0.63, 1.25, 2.5, 5, and 10 µM, each in triplicate.

### 2.3. Dataset Preparation

**Figure 1:**
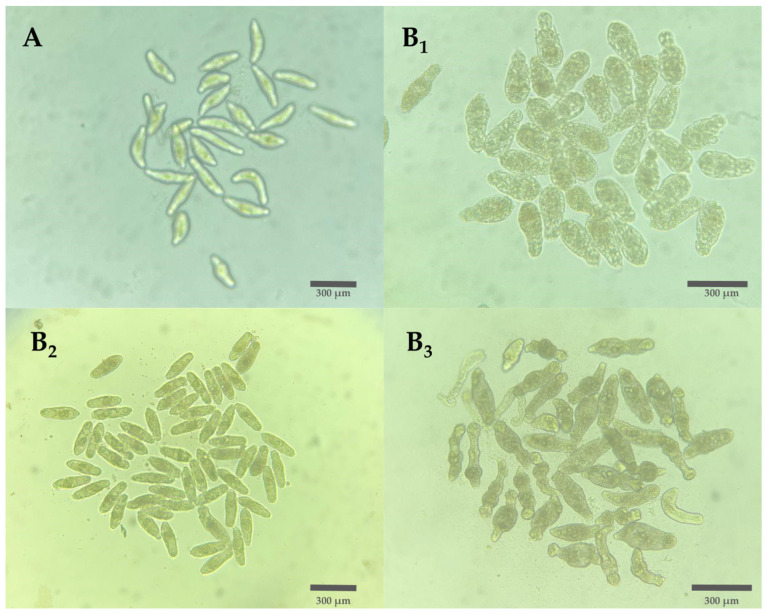
Morphology of healthy and damaged schistosomula in culture. (**A**) Healthy schistosomula with well-defined shape, refringent appearance, and structured internal body. (**B_1_**–**B_3_**) Predominantly damaged schistosomula displaying an irregular shape, granularity, and dark color, indicating tegmental and internal damage.

All images of the well bottoms were captured using a Redmi 7 mobile phone (Xiaomi, Haidian, Beijing, China) and an inverted bright field microscope (Eclipse Ts2, Nikon, Tokyo, Japan). The AxiWorm robotic arm, an adaptation of the AxiDraw minikit (Evil Mad Scientist, Sunnyvale, CA, USA), was used to scan the images in the 96-well plate [31]. The image collection comprised images from the controls, the antiparasitic panel, and compounds designed to target the colchicine-binding site. RoboFlow v1.0 software, was used to upload the photos, label schistosomula, organize them into datasets, and perform preprocessing tasks [24]. Schistosomula were classified into two categories based on phenotypic characteristics. The “Healthy” category included schistosomula with a defined shape, refringent appearance, and structured internal body, indicating viability. The “Damaged” category included those with an irregular shape, granularity, and dark color, indicating tegument and internal damage, which compromised their viability (Figure 1) [30]. Motility assessment of schistosomula was not considered. Manual annotation of schistosomula was performed using grids to assign them to one of two classes. Image augmentation techniques were also employed to generate variations from a single image. This process involved random application of saturation filters ranging from −25% to 25%, adjusting brightness between −15% and 15%, and adding a blur of up to 5 pixels. All images were standardized to 640 × 640 pixels in grayscale. After compiling a substantial dataset, uniform preprocessing was applied to all images. The dataset was split into training, validation, and test sets with respective ratios of 85%, 10%, and 5%.

### 2.4. YOLOv5 Model Training Pipeline

**Figure 2:**
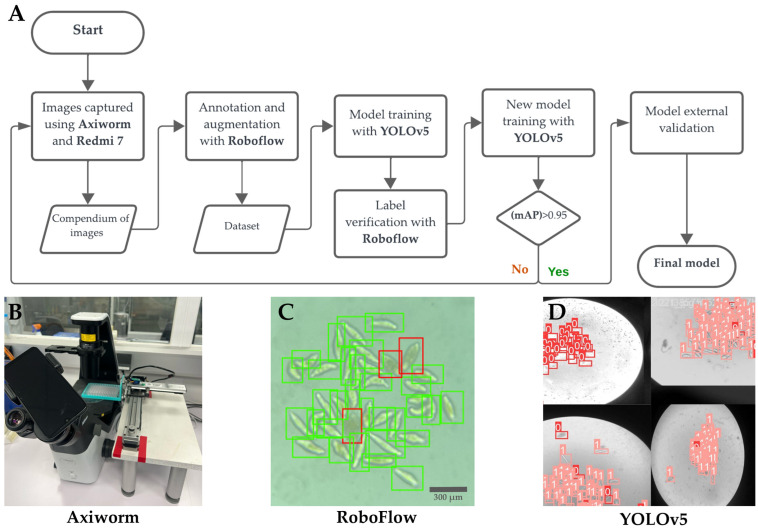
(**A**) Methods flowchart. Symbols: oval (start or end), rectangle (process), parallelogram (input/output), diamond (decision). (**B**) AxiWorm minikit. (**C**) RoboFlow software class annotation: damaged (red), healthy (green) (**D**) YOLOv5 model’s output predictions (0: damage, 1: healthy).

The YOLOv5 v7.0 (Ultralytics LLC, Frederick, MD, USA) object detection model was trained using Google Colab (Google LLC, Mountain View, CA, USA), a cloud-based platform that provides GPU support for enhanced processing speed. The training was conducted in the YOLOv5 framework, using an image size of 640 × 640 pixels, a batch size of 16, and 1000 epochs. Early stopping was applied with a patience of 30, meaning that training would stop if there was no improvement after 30 epochs [25]. Once the model achieved a mAP 0.5 (>0.7), it underwent model-assisted semi-automatic training. The model used the best-performing results from the previous training session, applying a confidence threshold of 0.4. After detecting the images, their annotations were uploaded to Roboflow for manual verification of the model’s predictions. Once verified, these images were incorporated back into the dataset, enabling further rounds of model-assisted semi-automatic training. This process was repeated multiple times with new images until the model reached the final mAP value (Figure 2). Mean average precision (mAP) is a primary metric for object detection precision, ranging from 0 to 1, with 1 indicating maximum accuracy. mAP calculation integrates precision, recall, and intersection over union (IOU). Precision denotes the model’s ability to correctly identify relevant objects, with a precision of 1.0 indicating zero false positives. IOU quantifies the overlap between model-predicted and annotated object outer edges (boundaries), ranging from 0.0 (no overlap) to 1.0 (exact match). Recall measures the model’s capability to detect all positive instances.

### 2.5. YOLOv5 Model External Validation Procedure

To validate the previously designed artificial intelligence model externally, 150 images were randomly selected from the *Schistosoma* image dataset. The model’s accuracy was assessed using two different confidence thresholds, 0.4 and 0.8. The model’s predictions were compared with those of an expert observer who initially labeled and corrected the data. This was made for the total count of schistosomula—healthy and damaged—and the viability percentage. To evaluate the model’s ability to accurately predict the lethal concentration for 50% (LC_50_), the prediction was assessed manually and using the model. The LC_50_ values were calculated using R programming language based on the viability data generated by the model and the expert observer [32]. Additionally, another external validation was performed by selecting 60 random images from the dataset. Three observers not involved in the model training conducted a manual count of healthy and damaged schistosomula three times. The YOLOv5 “detect” function was also applied to the same set of images for automated counting, using a confidence threshold of 0.4. The time taken by each observer to count and identify the 60 images was recorded. Absolute counting differences among the three manual counts and the model counts were analyzed using boxplots in R. The results from both manual counting and AI-based counting were compared using the Bland–Altman differences plot to validate the accuracy of the AI model. The Bland–Altman method assesses significant differences between manual and AI model counts, allowing comparison of two measurement techniques on the same quantitative variable. The total count and viability differences were calculated as YOLOv5 counting minus human counting.

### 2.6. Assay Application Through Screening of a Compound Library Procedure

To validate the model for a larger-scale assay, 186 compounds, primarily sulfonamides, benzimidazole derivatives, and other colchicine-binding site ligands (anti-tubulin compounds), were screened. Their putative mechanism of action involves interfering with tubulin polymerization by binding at the colchicine site. The screening process followed the methodology outlined in Section 2.2, with a final drug concentration of 10 µM for all compounds. LC_50_ values were calculated using the established in Section 2.2. PZQ was excluded as a control due to its ineffectiveness against the schistosomula stage, resulting from differences in target molecule expression and stage-specific physiology [33]. Therefore, Auranofin, an effective control in schistosomula drug-sensitivity assays and shown to have activity against *S. mansoni*, was selected for our study [30,34].

## 3. Results

### 3.1. YOLOv5 Model Training

**Figure 3:**
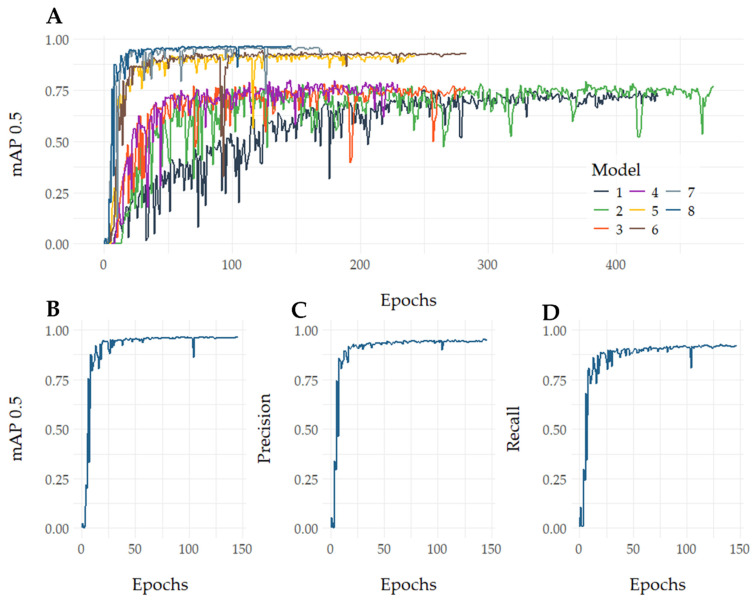
Performance evaluation metrics for schistosomula detection models. (**A**) Comparative performance evaluation of eight models. (**B**) mAP 0.5 score for Model 8. (**C**) Precision metric for Model 8. (**D**) Recall metric for Model 8.

A total of 4390 images were obtained, containing 715 null examples, resulting in 27,639 total labels. Eight models were trained using the same hyperparameters without pre-trained weights. The number of images used for each model was as follows: 553 images for the first and second models, 945 for the third model, 1032 for the fourth, 1392 for the fifth, 1674 for the sixth, and 2531 for the seventh. The eighth model, trained with 4390 images over 146 epochs, achieved a mean average precision (mAP) of 0.966 (Figure 3).

**Figure 4:**
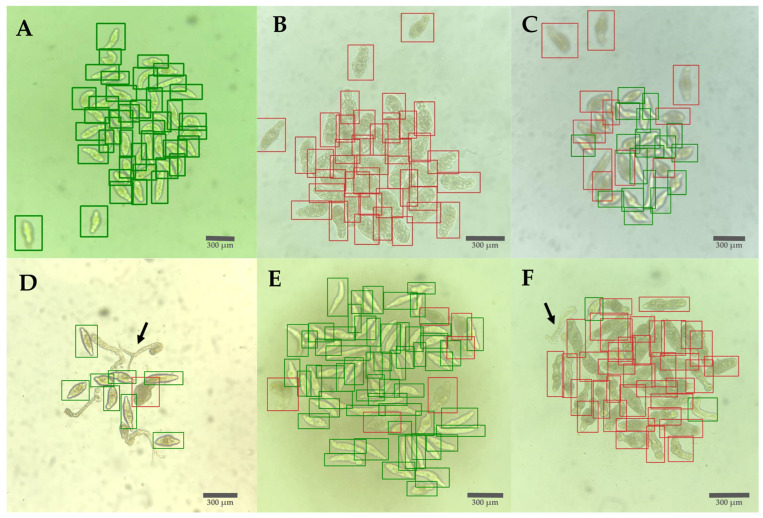
Detection of schistosomula using YOLOv5 showing different morphologies under different conditions after 72 h treatment: damaged (red), healthy (green): (**A**) Healthy schistosomula. (**B**) Schistosomula with high granularity. (**C**) Mixed healthy and damaged schistosomula. (**D**) Arrows indicating untransformed cercaria and tails are excluded. (**E**) Schistosomula with high contrast. (**F**) Predominantly damaged schistosomula with evident deformation.

The internal validation obtained a precision value of 0.946 and a recall value of 0.917. When verifying the model labels, it was found that the model is versatile and accurate in identifying schistosomula in different situations. The YOLOv5 model identified healthy schistosomula in control wells (Figure 4A), damaged schistosomula exhibiting internal granularity, with or without tegument deformities (Figure 4B,F), and mixtures of healthy and damaged schistosomula (Figure 4C–E). It also successfully excluded non-transformed cercariae and their tails (Figure 4D,F) and detected schistosomula in high-contrast images (Figure 4E).

### 3.2. YOLOv5 Model External Validation

**Figure 5:**
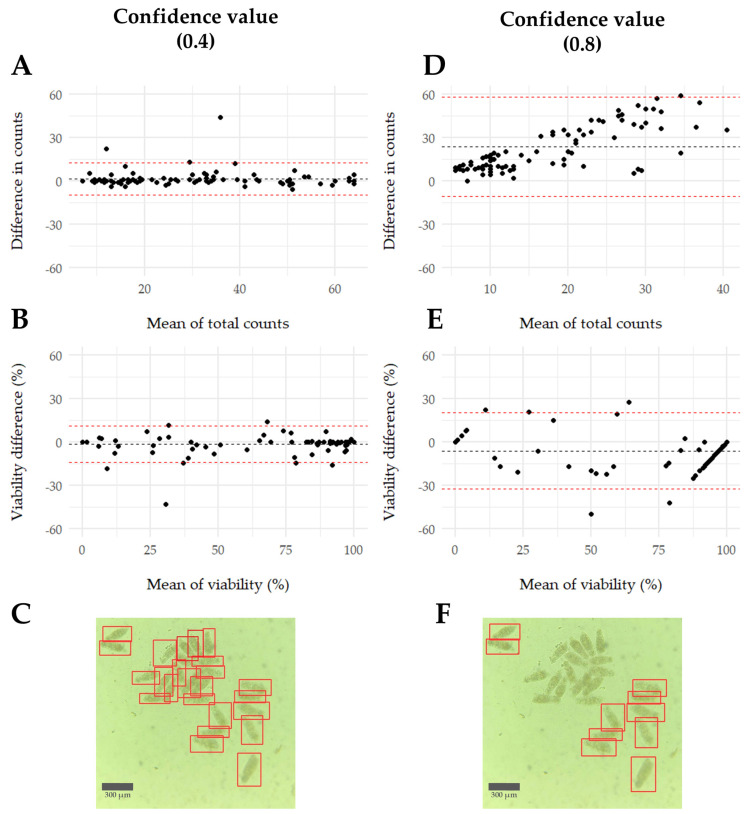
Bland–Altman model evaluation (YOLOv5 vs. manual counting) at different confidence values. (**A**) Total count difference at 0.4 confidence threshold. (**B**) Viability percentage difference at 0.4 confidence threshold. (**C**) YOLOv5 prediction at 0.4 confidence threshold. Damaged schistosomula are inside red boxes. (**D**) Total count difference at 0.8 confidence threshold. (**E**) Viability percentage difference at 0.8 confidence threshold. (**F**) YOLOv5 prediction at 0.8 confidence threshold. Damaged schistosomula are inside red boxes All differences were calculated = (YOLOv5 counting − human counting). Means are shown in black dot lines and standard deviations shown in red dot lines.

Results from the Bland–Altman analysis indicated that the model performed better with a confidence threshold of 0.4 compared to 0.8. This was evidenced by a mean closer to 0 and a larger proportion of data points within the standard deviation. The standard deviations increased at the 0.8 confidence threshold compared to the 0.4 threshold. Specifically, the standard deviations were 13.50 and 6.35 for viability and 17.46 and 5.60 for total count, respectively. Differences were more noticeable in total counts than in viability percentages. Additionally, as observed in Figure 5F, using a higher confidence value, some schistosomula were not counted, whereas Figure 5C shows that most if not all, were included (Figure 5). 

Moreover, similar concentration values were obtained with both manual and YOLOv5 model preliminary LC_50_ calculations. For Auranofin, YOLOv5 calculated an LC_50_ of 0.14 μM ± 0.01, while the human calculation resulted in an LC_50_ of 0.13 ± 0.01 μM. For Anti-tubulin Compound 1, YOLOv5 determined an LC_50_ of 1.91 ± 0.16 μM, while the human calculation resulted in 1.81 ± 0.09 μM. Lastly, for anti-tubulin Compound 2, YOLOv5 produced an LC_50_ of 3.04 ± 0.06 μM, compared to 3.06 ± 0.03 μM from human assessment.

**Figure 6:**
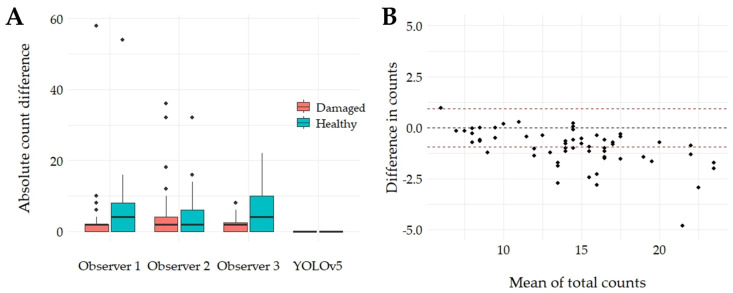
Differences between YOLOv5 and manual counting by counters who were not involved in the model training. (**A**) Absolute manual count difference (between three counts). (**B**) Difference in counts: Bland–Altman plot, means are in black dot line and standard deviations are in red dot lines.

Furthermore, the differences among the three observers showed variations in their counts of schistosomula, including both damaged and healthy specimens, as well as the total count (Figure 6A). Some counts varied significantly, with discrepancies of up to 60 between observers, highlighting the variability in their assessments. In contrast, the YOLOv5 model consistently counted the same number across all three observations, with an average difference of 0 (Figure 6A). The mean time taken by the first observer was 14.7 min, 80.8 min for the second, and 20.4 min for the last observer, with an average of 38.6 min. Meanwhile, YOLOv5 detection was completed in less than a minute. The recorded time only includes counting the pre-taken photographs and excludes the time for manually moving and assessing each plate under the microscope. This method was chosen to prevent a reduction in schistosomula viability, which could occur if different observers manually counted the same plates three times during independent visualizations. This approach ensures consistent viability assessment across all counts. Complete manual counting by an expert counter without the assistance of AxiWorm and YOLOv5 can take between 30 to 40 min, plus additional data analysis time. The study of differences among observers using the Bland–Altman plot revealed significant differences between the means of the three observers and the model, particularly in the difference in counts, with many outliers beyond the standard deviation (Figure 6B).

### 3.3. Assay Application Through Screening of a Compound Library

**Figure 7:**
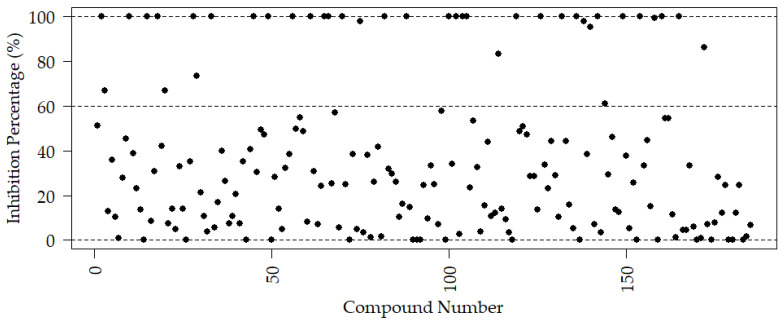
Percentage of inhibition of the anti-tubulin compound library at 72 h. A > 60% inhibition threshold corresponds to moderate activity, while a 100% threshold corresponds to positive activity, marked in a dot line.

**Figure 8:**
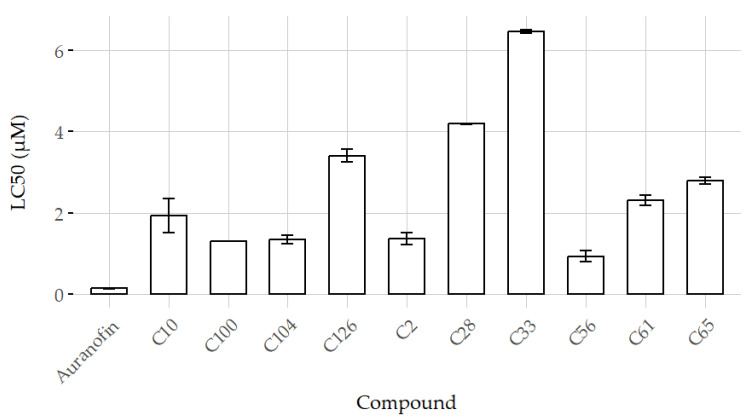
Ten preliminary LC_50_ values of active compounds identified through AI-assisted screening assay.

Of the 185 anti-tubulin compounds screened using this assay, 28 showed positive activity with 100% inhibition at 72 h. Ten additional compounds exhibited mild to moderate activity, with more than 60% inhibition at 72 h (Figure 7). From the active compounds at a final concentration of 10 µM, 15 compounds exhibited LC_50_ ranging from 0.90 µM to 6.5 µM, whereas the remaining compounds had LC_50_ values between 6.6 µM and 10 µM. Ten of these compounds are presented in Figure 8.

## 4. Discussion

The anthelmintic drug PZQ, licensed in the mid-1980s, has been effective in schistosomiasis treatment and supporting mass control campaigns for over 40 years. However, it does not eliminate the juvenile stages (schistosomula) of parasites or prevent reinfections, and concerns about emerging resistance have been growing after decades of widespread use. Consequently, control programs relying only on this drug achieve temporary effects. Therefore, new drug targets and drug repositioning are being proposed, requiring the development of high-performance assessment techniques [35]. This study presents a novel method for assessing schistosomula viability, using YOLOv5 software for object identification to distinguish between healthy and damaged schistosomula, which can be integrated into drug screening pipelines. This technique could assess new drug activity against *S. mansoni* schistosomula at different time points without disturbing the parasite culture. Moreover, it eliminates the need for specialized devices such as fluorescence microscopes or invasive techniques measuring metabolism or staining properties compromising the normal schistosomula behavior. Instead, this method uses a simple inverted bright field microscope, a customized app, and a smartphone camera assisted by a robotic arm. Moreover, the YOLOv5 model could offer accuracy, repeatability, and tractability in the assessment records.

This new image recognition AI model achieved an overall average mAP of 0.966 using 4390 images. These values suggest that the model is highly precise and reliable in its overall classification performance. Achieving such high accuracy and mAP with a middle-sized dataset demonstrates the model’s robust design and effective training. Comparable models with similar mAP and precision have been published. For instance, in the study by Tian et al. [36], a mAP of 0.932 was reported for a detection system of dorsal hand veins, and Jubayer et al. [37] achieved a mAP of 0.966 for detecting mold on food surfaces. In parasitology, other studies have shown similar precision, such as 0.952 in recognizing *Plasmodium* spp. stages [27] or with an average mAP of 0.970 in detecting intestinal parasite eggs with YOLOv5 [38]. The model also accurately identifies schistosomula in varied conditions, including deformities and excretory compound contrasts, and prevents cercaria misclassification. Several models were trained to achieve a higher mean average precision (mAP). Each new model showed improved mAP, eventually reaching a top 0.966 value, up from 0.75 in the earlier models. This improvement was directly attributed to the increased number of images, as more images directly correlate with the model’s reliability. Maintenance cycles stop at 146 to avoid over-adjustment since no improvement was detected. The software ends training if no progress is observed over 30 cycles (epochs), ensuring optimal performance and preventing overfitting, which limits its ability on new or different data scenarios [39].

The Bland–Altman analysis showed that the last model performs better with a confidence level of 0.4 compared to 0.8. The mean differences were minimal, with most data points falling within the standard deviation, especially in the total count mean and viability percentage. Lowering confidence increases false positives but decreases false negatives. Since the schistosomula cultures are clean and rarely contain contamination, the minimal false positives make it more beneficial to focus on reducing false negatives. Thus, these findings suggest that adopting a lower confidence level enhances performance while ensuring consistency, unlike higher confidence levels. The trained model also proved useful for drug screening, consistently providing similar results in calculating LC_50_ values. This indicates that the model reliably evaluates the effectiveness of new compounds for potential therapeutic use. Similar machine learning systems have been applied to measure the minimum inhibitory concentration (MIC) in bacterial isolates [40] and to determine the median effective concentration (EC_50_) using *Daphnia magna* [41].

Traditional microscopy methods for assessing parasite viability are labor-intensive, time-consuming, and subjective due to their reliance on human observers, requiring qualified personnel to ensure accurate results [23]. Conventional techniques to assess *S. mansoni* schistosomula involve trained personnel. Previously, it took up to 40 min to fully evaluate a 96-well plate by manually examining each plate under a microscope to assess schistosomula viability. With YOLOv5 trained on our dataset, detecting, classifying, and counting schistosomula across 60 photographs now takes less than 1 min. This significantly improves the average human detection time for the same task. This reduction in detection time helps minimize the period for which plates stay outside the incubator. This aligns with protocols such as those by Lombardo et al. [30], which recommend a maximum of 15 min outside the incubator. Additionally, observer bias must be considered, as different observers may introduce variability in schistosomula counts even when the same images are counted repeatedly by various observers. The external validation involving three observers highlighted this issue, where despite each observer counting the images three times, differences (up to 60) in counts persisted. Additionally, variations were noted in counts of identical images that were expected to be consistent among all observers. In contrast, the YOLOv5 model ensured consistent and accurate counting across the dataset, eliminating the inconsistencies associated with human subjectivity. This automated approach enhances the reliability and reproducibility of viability assessments and standardizes the process, improving overall efficiency and reducing potential errors in data interpretation as a result [20].

The recent screening of 186 anti-tubulin compounds using the novel method for assessing schistosomula viability has produced promising results, demonstrating the effectiveness of this approach in drug discovery. Among the screened compounds, 31 inhibited 100% at 72 h, indicating strong anti-parasitic activity. Additionally, 12 compounds showed mild to moderate activity with inhibitions over 70%. The method’s precision facilitated the determination of LC_50_ values, with 15 compounds ranging from 0.9 to 6.5 µM, highlighting their potential therapeutic efficacy.

The present model also has limitations, as it performs the analysis on photographs and cannot detect the effects of compounds on the motility of the worms. While phenotype algorithms usually work well to distinguish damaged from undamaged schistosomula, combining phenotype and motility analysis can provide even more precise discrimination [23]. As a next step, it would be interesting to adapt the present model to differentiate the motility of schistosomula, thereby enhancing the system’s reliability. However, the current approach is well-suited for initial compound screening.

## 5. Conclusions

The artificial intelligence model effectively differentiated between healthy and damaged schistosomula, achieving an average mAP of 0.966 (96.6%). This AI-powered method significantly improves accuracy and time efficiency compared to traditional microscopy, enabling the evaluation of compounds for potential schistosomiasis drugs without the need for dyes or specialized equipment, thus facilitating more efficient drug assessment. Additionally, it could be adapted and further developed for larger-scale assays in the future. The screening assay, powered by an AI model, successfully identified 38 potential compounds with schistosomicidal activity. Among these, 28 compounds demonstrated complete inhibition after 72 h of exposure. Further studies, including in vivo assays, are required to validate these compounds and others evaluated through our model to determine their true therapeutic potential.

## Data Availability

The images used in this study were generated by the authors. Annotations for these images were performed using Roboflow, and the YOLOv5 model was trained on this annotated dataset. The annotated dataset is available upon request from the authors. The code and configurations for the YOLOv5 model can be found in the GitHub repository at https://github.com/ultralytics/yolov5, accessed on 20 December 2023.
